# Repurposed Effect of ^177^Lu-DOTATATE in the Treatment of Mantle Cell Lymphoma

**DOI:** 10.3390/curroncol29100594

**Published:** 2022-10-09

**Authors:** Mohamad K. Elajami, Lorena P. Burton, Hisham F. Bahmad, Gerard Chaaya, Michael Schwartz

**Affiliations:** 1 Department of Internal Medicine, Mount Sinai Medical Center, Miami Beach, FL 33140, USA; 2 Arkadi M. Rywlin M.D. Department of Pathology and Laboratory Medicine, Mount Sinai Medical Center, Miami Beach, FL 33140, USA; 3 Department of Internal Medicine, Division of Hematology and Oncology, Mount Sinai Medical Center, Miami Beach, FL 33140, USA

**Keywords:** DOTATATE, mantle cell lymphoma, carcinoid, drug repurposing, case report

## Abstract

Mantle cell lymphoma (MCL) is an uncommon subcategory of non-Hodgkin lymphoma (NHL). Pathogenesis primarily includes overexpression of *CCND1* and *SOX11* along with other molecular aberrations. Lutetium ^177^Lu-DOTATATE is a radiolabeled somatostatin analogue used for the treatment of gastrointestinal neuroendocrine tumors. There are no clinical data supporting the use of Lutetium ^177^Lu-DOTATATE in the treatment of lymphoma. We describe the case of an 84-year-old man with a history of MCL and carcinoid tumor of the lung. Following progression of the carcinoid malignancy, the patient was treated with Lutetium ^177^Lu-DOTATATE. After treatment, there was an overall improvement of the patient’s MCL that was demonstrated by stable lymphadenopathy on serial CT scans and down-trend of the absolute lymphocyte count. Therefore, we hypothesize that ^177^Lu-DOTATATE might have a role and can be repurposed for treating MCL.

## 1. Introduction

Mantle cell lymphoma (MCL) is a less common subcategory of B-cell non-Hodgkin lymphoma (NHL). It accounts for 3–10% of NHL cases in adults with persistently rising incidence [1]. Pathogenesis commonly involves *CCND1* and *SOX11* overexpression and *TP53* mutations, as well as other less common genetic aberrations and molecular signatures [2]. The mainstay for treatment in fit patients diagnosed with MCL includes cytarabine-based induction chemotherapy followed by autologous stem cell transplant (ASCT) and maintenance therapy with Rituximab (anti CD20 monoclonal antibody) [3,4].

Lutetium ^177^Lu-DOTATATE is a radiolabeled somatostatin analogue that binds to somatostatin receptors, mainly subtype 2 receptors [5]. It was approved by the U.S. food and drug administration (FDA) in 2018 for the treatment of foregut, midgut, and hindgut neuroendocrine tumors (NETTER-1 trial) [6]. In addition to treatment of neuroendocrine tumors, this drug is under clinical trials assessing its anti-tumor effects in meningioma (*NCT03971461* and *NCT04082520*; ClinicalTrials.gov), glioblastoma (*NCT05109728*; ClinicalTrials.gov), neuroblastoma (*NCT04903899*, *NCT03966651*; ClinicalTrials.gov), pheochromocytoma and paraganglioma (*NCT04106843*, *NCT03923257*; ClinicalTrials.gov). However, to our knowledge, there are no studies in the literature that tackle the effect of Lutetium ^177^Lu-DOTATATE in lymphoma. 

We describe the case of an 84-year-old man with a history of typical carcinoid tumor of the right lower lung lobe, for which he underwent a lobectomy, and indolent leukemic MCL. The patient experienced recurrence of the carcinoid tumor in the left upper lung lobe that was unresponsive to Sandostatin. Accordingly, he underwent four cycles of ^177^Lu-DOTATATE and, unexpectedly, he had an overall improvement of his MCL. This case report was conducted and reported in accordance with CAse REports (CARE) guidelines for reporting case reports.

## 2. Case Presentation

An 84-year-old man with a history of carcinoid tumor of the lung, leukemic mantle cell lymphoma (MCL), prediabetes, meningitis (in 1980) with secondary seizure disorder (last episode in 1994), and benign prostatic hyperplasia, presented to the oncology clinic of our cancer center for follow-up on his lymphoma. The patient had a smoking history of two packs per day for 10 years but quit 50 years ago. His body mass index (BMI) was 29.03 kg/m². The patient had a known allergy to barbiturates and midazolam. 

He initially presented in 2013 with a chief complaint of hematuria. Computerized tomography (CT) of the abdomen and pelvis revealed renal stones and was incidentally notable for a right lower lobe (RLL) lung mass. Further imaging with CT of the chest confirmed the presence of a 1.8 cm mass as well as a 0.7 cm left upper lobe (LUL) nodule. A positron emission tomography (PET)-CT scan of the chest demonstrated hypermetabolic uptake in the RLL lesion. The patient was referred to a cardiothoracic surgeon and underwent a robotic video-assisted thoracoscopy with right lower lobectomy and mediastinal lymphadenectomy. The intraoperative and postoperative course were uncomplicated. Histopathologic workup of the resected lesion demonstrated a 3.2 cm typical carcinoid tumor with solitary lymph node involvement, clinical stage IIA (pT2aN1Mx). Hematoxylin and eosin (H&E)-stained sections revealed a neoplastic proliferation of cells forming nests, with round nuclei, stippled chromatin, and scant cytoplasm. No evidence of necrosis was identified. Diagnosis of typical carcinoid tumor was given. Immunohistochemical stains were positive for chromogranin and synaptophysin (neuroendocrine cell markers), CD56, CAM5.2, and CK7 (epithelial cell markers), while negative for CK20 in the tumor cells. A Ki-67 showed a low proliferative index with staining in approximately 1% of the cells. The IHC stains confirmed the diagnosis of typical carcinoid tumor. The patient did not receive adjuvant therapy due to low-grade tumor and no evidence of recurrence. 

The patient had been also diagnosed with early-stage chronic lymphocytic leukemia (CLL) in 2013. He had been managed conservatively with serial complete blood counts with deferential (CBCD) as chemotherapy was not indicated at that time. However, and for further evaluation of CLL, fluorescence in-situ hybridization (FISH) of the peripheral blood revealed trisomy 12 in 26.5% of the analyzed nuclei and abnormal fusion signals of *CCND1(BCL1)/IgH* fusion; t(11;14) (q13;q32), suggestive of indolent leukemic phase MCL. A repeat PET-CT scan of the chest in 2018 demonstrated an increase in the size of the LUL nodule to 2.0 cm. The patient was asymptomatic, and no further surgical intervention was indicated.

**Figure 1:**
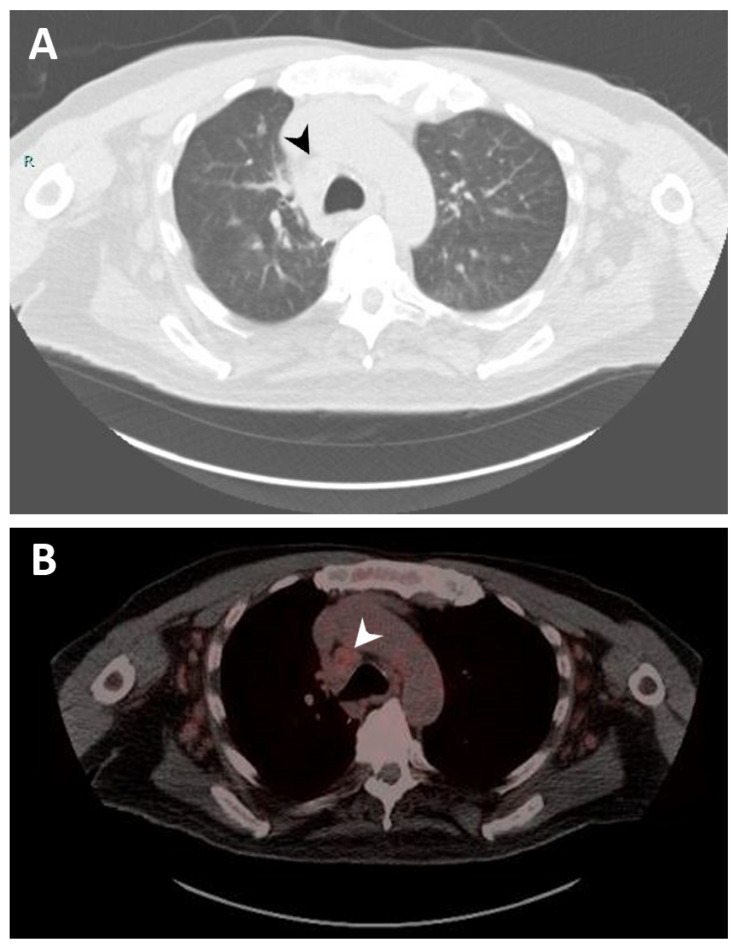
Imaging studies. (**A**) Computerized tomography (CT) scan of the chest showing right lower paratracheal lymphadenopathy measuring 2.1 cm (black arrow). (**B**) Gallium-68 dotatate positron emission tomography (PET)/CT scan of the chest demonstrating right lower paratracheal lymphadenopathy with mild radiotracer uptake, suggesting a neoplastic process related to mantle cell lymphoma (white arrow).

In 2020, the patient was admitted to the hospital due to progressive shortness of breath. Imaging reported increased LUL mass size to 5.9 cm encasing the bronchovascular bundle of the lingula. A biopsy of the specimen reported neoplastic cells were strongly and diffusely immunoreactive for synaptophysin, confirming the diagnosis of typical carcinoid tumor. CT of the chest also showed extensive lymphadenopathy throughout the neck, chest, and upper abdomen (Figure 1A). The patient was started on octreotide acetate (Sandostatin) and everolimus for typical carcinoid tumor. However, everolimus was stopped due to development of side effects (stomatitis and nausea). Octreotide acetate was continued. A gallium-68 dotatate PET/CT scan was performed to identify the presence of neuroendocrine tumor in the lymph nodes, and it showed moderate to intense activity in the lung and a low level of activity in mediastinal lymph nodes indicating lymph node from MCL (Figure 1B). Later, a paratracheal lymph nodes biopsy confirmed MCL. The patient continued with octreotide acetate for the following year. As the carcinoid disease showed progression, the patient was a candidate for treatment with ^177^Lu-DOTATATE in combination with Sandostatin. He received four cycles of ^177^Lu-DOTATATE. Follow-up imaging with a serial CT scan of the chest throughout the clinical course until present showed no progression of the preexisting pulmonary nodules and lymphadenopathy. In addition, follow-up with serial CBCD showed a selective down-trend of the absolute lymphocyte count from 20,970 to 2200 per μL (reference range 1200–4000 per μL).

Nevertheless, the patient was admitted again to our institution in August 2022 for progressive shortness of breath. He tested positive for SARS-CoV-2 and was diagnosed with COVID pneumonia. During hospitalization, the patient continued to decline and was referred to hospice care as per the family’s request. The patient expired in September 2022.

## 3. Discussion

Mantle cell lymphoma (MCL) is a rare and aggressive subcategory of non-Hodgkin lymphoma (NHL), accounting for 3–10% of NHL cases [1]. Predominantly studied pathogenesis involves *CCND1* and *SOX11* overexpression as well as *TP53* mutations [7,8,9]. The standard of care treatment in patients with newly diagnosed MCL is comprised of involved field radiotherapy (IF-RT) in stages I or II of the disease and induction chemotherapy with cytarabine followed by autologous stem-cell transplantation and maintenance therapy with Rituximab in fit patients presenting with stage III or IV disease [3,4]. In contradiction, radioimmunotherapy, which combines an antibody with radionuclide to deliver cytotoxic radiation to target tissues, has shown efficacy in NHL [10,11]. Two anti-CD20 monoclonal antibodies were approved by the FDA in early 2000s for the treatment of NHL but have not been widely implemented in the clinical practice: ^90^Y-ibritumomab tiuxetan and ^131^I-tositumomab [12]. 

A newer treatment paradigm for patients with inoperable and metastasized gastroenteropancreatic neuroendocrine tumors (GEPNETs) includes the use of peptide receptor radionuclide therapy (PRRT) [13]. ^177^Lu-DOTATATE, an example of PRRT, was approved by the FDA in 2018 for the treatment of somatostatin receptor-positive GEPNETs through its higher affinity to somatostatin subtype-2 receptors [6]. It is a combined radiopharmacological medication that consists of the radionuclide Lu-177 and the somatostatin analogue DOTATATE. Lu-177 is a medium energy beta emitter and a low energy gamma emitter which yields ionizing radiation exclusively to the tumor cells creating single- and double-strand DNA breaks which eventually lead to cell death [14].

Drug repurposing is a propitious approach in treating various diseases, including cancer. It involves the use of medications, previously approved by the FDA, for a newer indication other than their traditional ones. Driven by the advancements in discovering the genetic and epigenetic alterations of tumor cells, this promising approach is expected to provide novel pharmacological agents that overpass the resistance to conventional therapy that is expressed in multiple cancers [15,16]. The repurposed effect of many medications has been studied in colorectal cancer [17], pancreatic cancer [18], and brain tumors [19,20]. Moreover, non-Hodgkin lymphoma has also been targeted in drug repurposing studies. Clarithromycin has shown promising results in patients with mucosa-associated lymphoid tissues (MALT) lymphoma with an overall response rate (ORR) of 52% and a 2-year progression free survival of 56% [21,22]. Another study by Han et al. demonstrated an antiproliferative activity in MCL cell lines using alisertib, carfilzomib, pracinostat, and YM155 [23].

Physiologically, there is a continuous crosstalk between the neuroendocrine and immune systems. Neuroendocrine hormones regulate lymphoid cell activity, including proliferation and mitogenesis, through an autocrine and paracrine effect on receptors shared by both systems [24,25]. Somatostatins have been shown to halt lymphocyte proliferation [26,27], endotoxin-mediated leukocytosis, IgA secretion by lymphocytes and express a regulatory effect on macrophages [28,29]. Although somatostatin receptors are expressed in most of the neuroendocrine neoplasms, it has been found in studies to occur in CNS [30], breast [31], and lung [32] tumors. Moreover, surgical biopsies of both B and T non-Hodgkin’s lymphoma and Hodgkin disease patients were remarkable for somatostatin receptors expression [33]. However, there is no clinical evidence that supports the use of somatostatin analogues in the treatment of non-Hodgkin lymphoma. 

In our presented case, we hypothesize that ^177^Lu-DOTATATE expresses a repurposed effect in treating MCL. Although there is no clinical data that support the use of ^177^Lu-DOTATATE in patients with MCL, we believe that the somatostatin component of this radiopharmacological medication exhibited an antiproliferative effect on lymphocytes and potentiated the effect of Lu-177 which in turn induced a single and double stranded DNA break, yielding tumor cell death. This paved the way for the pivotal role of a targeted radiation effect, potentiated by peptide receptor analogue, in treating early-stage MCL.

Somatostatin receptors are heterogeneously expressed among various lymphoma subtypes, but their clinical significance is not established, and their expression is generally at a lower level compared to neuroendocrine tumors [24,33]. Multiple studies demonstrated somatostatin receptor expression in lymphomatous tissue, predominantly diffuse large B-cell lymphoma, follicular lymphoma, and Hodgkin lymphoma, but very low expression was notable in patients with MCL, and mucosa associated marginal B-cell lymphoma (MALT) [24,34]. Somatostatin receptor expression in lymphomatous tissue may serve as a potential therapeutic target for novel peptide receptor radionuclide therapy which in turn alters the prognosis of low-grade lymphoma, preventing disease progression. Nevertheless, it is still unclear whether the presence of peptide receptors, other than somatostatin receptors, within the lymph nodes increase the lymphomatous tissue affinity to ^177^Lu-DOTATATE.

## Data Availability

Not applicable.
